# STrengthening the Reporting of OBservational studies in Epidemiology – Molecular Epidemiology (STROBE-ME): An Extension of the STROBE Statement

**DOI:** 10.1371/journal.pmed.1001117

**Published:** 2011-10-25

**Authors:** Valentina Gallo, Matthias Egger, Valerie McCormack, Peter B. Farmer, John P. A. Ioannidis, Micheline Kirsch-Volders, Giuseppe Matullo, David H. Phillips, Bernadette Schoket, Ulf Stromberg, Roel Vermeulen, Christopher Wild, Miquel Porta, Paolo Vineis

**Affiliations:** 1 Department of Epidemiology and Biostatistics, School of Public Health, Imperial College London, London, United Kingdom; 2 Social and Environmental Health Research, London School of Hygiene and Tropical Medicine, London, United Kingdom; 3 Institute of Social and Preventive Medicine, University of Bern, Bern, Switzerland; 4 International Agency for Research on Cancer (IARC) Lyon, France; 5 Department of Cancer Studies and Molecular Medicine, University of Leicester, Leicester, United Kingdom; 6 Stanford Prevention Research Centre, Stanford University School of Medicine, Stanford, California, United States of America; 7 Department of Hygiene and Epidemiology, University of Ioannina School of Medicine, Ioannina, Greece; 8 Laboratory for Cell Genetics, Vrije Universiteit Brussel, Brussels, Belgium; 9 HuGeF Human Genetics Foundation, Turin, Italy; 10 Department of Genetics, Biology and Biochemistry, University of Turin, Turin, Italy; 11 Institute of Cancer Research, Sutton, United Kingdom; 12 National Institute of Environmental Health, Budapest, Hungary; 13 Division of Occupational and Environmental Medicine, Lund University, Lund, Sweden; 14 Institute for Risk Assessment Sciences (IRAS), Division Environmental Epidemiology, Utrecht University, Utrecht, the Netherlands; 15 Institut Municipal d'Investigacio Medica (IMIM), Universitat Autonoma de Barcelona, Barcelona, Spain; 16 MRC-HPA Centre for Environment and Health, School of Public Health, Imperial College London, London, United Kingdom

## Abstract

Valentina Gallo and colleagues provide detailed guidance to authors to help more accurately report the findings of epidemiological studies involving biomarkers. Their guidance covers issues regarding collection, handling and storage of biological samples; laboratory methods, validity and reliability of biomarkers; specificities of study design; and ethical considerations.

**Summary Points**

- Advances in laboratory techniques have led to the increasing use of biomarkers in epidemiological studies, but the quality of reporting of such studies varies.
- The STROBE (STrengthening Reporting of Observational studies in Epidemiology) initiative, established in 2004, provides guidance on reporting observational epidemiology studies.
- Here, the STROBE-ME (Strengthening the Reporting of OBservational studies in Epidemiology – Molecular Epidemiology) initiative builds on STROBE and provides additional guidance on reporting biomarker studies.
- Specific additions relate to the collection, handling and storage of biological samples; laboratory methods, validity and reliability of biomarkers; specificities of study design; and ethical considerations.
- A checklist to help authors in reporting biomarker studies is published as supporting information (Table S1).

## Introduction

**Figure 1:**
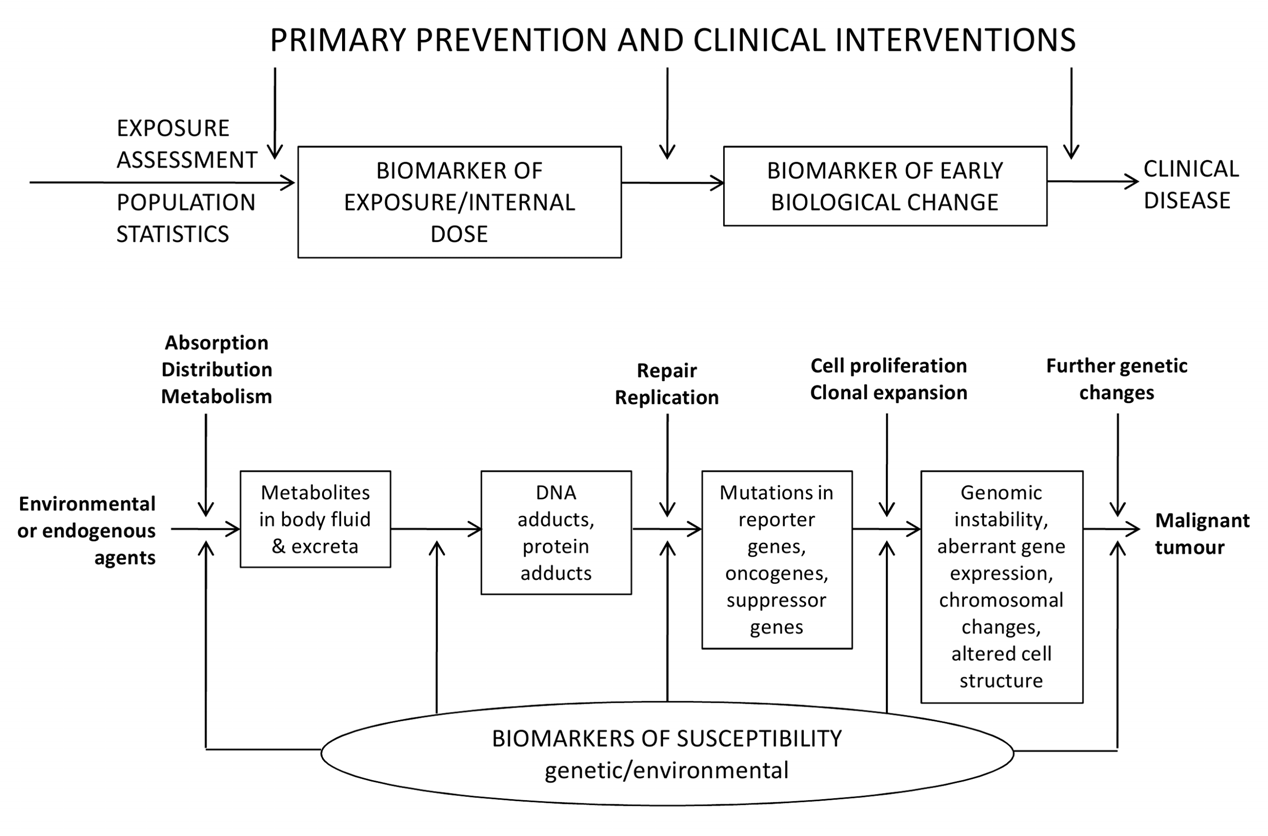
Schematic framework on the use of biomarkers in molecular epidemiology studies. Adapted from Vineis and Perera [42].

In recent years, advances in laboratory techniques have led to a rapidly increasing use of biomarkers in epidemiological studies, a field known as *molecular epidemiology* [1]–[5]. Biomarkers are any substance, structure or process that can be measured in biospecimens and may be associated with health-related outcomes. Biomarkers of internal dose, of early biological change and of susceptibility (see Figure 1 and Box 1 for definitions) are used as proxies for investigating the interplay between external and/or endogenous agents and the body. Biomarkers may provide valuable scientific tools because of their ability to inform biological mechanisms through the examination of early, intermediate and late molecular and cellular events. Moreover, a biomarker may capture several external exposure variables in a single biologically relevant quantity, provide quantitative measurements, increase statistical power or be used as an efficient and informative intermediate outcome. Finally, biomarkers can be used to identify susceptible individuals and to improve diagnosis and early detection of disease as well as prediction of major clinical outcomes in patients with a given disease. Figure 1 describes the whole spectrum of applications of biomarkers; the scheme uses cancer as an example because this is the field in which the conceptual framework of molecular epidemiology has had the greatest development and numerous postulated potential applications; however, similar concepts apply to many other fields.

**Box 1. Definitions of Terms Used in the Text**

There are several definitions of biomarkers. The most commonly adopted states that a biomarker is any substance or biological structure that can be measured in the human body and may influence, explain or predict the incidence or outcome of disease [24]. According to another definition, a biomarker is ‘a characteristic that is objectively measured and evaluated as an indicator of normal biological processes, pathogenic processes or pharmacologic responses to a therapeutic intervention’ [43]. Biomarkers are measured in human biospecimens typically using molecular, biochemical and cytogenetic techniques. Some investigators also include under the biomarker umbrella measures derived from modern imaging techniques that aim to characterize biological process, e.g. from positron emission tomography or functional magnetic resonance imaging. However, these biomarkers also entail issues that are specific to image processing and interpretation that are beyond the scope of the guidance provided in this manuscript. Some biomarkers (but not ‘exposure biomarkers’) allow insight into the cellular processes in the human body and serve to explore the links among environmental/endogenous exposures, the genome, host factors/structures and disease. Based on the concept that there is continuity between exposure to an external agent, its metabolism within the body and the onset of a resulting time-delayed disease, we can distinguish three main types of biomarkers that are able to investigate the internal process of interaction between the external agent and the body (Figure 1).

A **biomarker of exposure/internal dose** is an indicator of current and/or past exposure to environmental agents. Biomarkers of internal dose may indicate, depending on their nature, a recent or very recent exposure as well as a long-term exposure. The ideal biomarker of exposure is specific, detectable at very low concentrations, in quantitative relationship with the level of exposure, and its levels integrate over time.

Metabolite concentrations change rapidly with a short half-life from a few hours up to a few days and may show a large daily intra-individual variation as well as inter-individual variation. They may be specific for certain exposures or integrate several types of exposure. For example, urinary 1-hydroxypyrene concentration is a surrogate for the measurement of complex PAH exposure via different exposure routes, whereas urinary 4-(methylnitrosamino)-1-(3-pyridinyl)-1-butanol (NNAL), a metabolite of 4-(methylnitrosamino)-1-(3-pyridinyl)-1-butanone (NNK), and its glucuronides are specific biomarkers of exposure to tobacco smoke. A wide variety of highly sensitive analytical methods are used for the detection of parent compounds and their metabolites in human biospecimens.

**Biomarkers of early biological change** are biomarkers that reflect the interaction between the external agent and the exposed body. They usually encompass a broad and heterogeneous category; their main advantage is that their presence in subjects is usually more frequent than the disease itself and they can be detected earlier, thus allowing researchers to identify potential harm before a clinical disease manifests. Biomarkers of early biological change include markers of early detection of disease and also prognostic markers if the outcome is death, recurrence or disability.

**Biomarkers of susceptibility** include multiple subcategories, which encompass both acquired (phenotypic) biomarkers and genotypic markers [2]. Examples of the former are biomarkers of previous disease, whereas genotypic markers include the more extensively studied category of inherited genetic variants. Concerning the latter, an essential issue is whether and how gene variants manifest themselves in cellular functions and phenotypes and how they influence individual susceptibility to environmental exposures. These include also cellular phenotypes (such as DNA repair capacity) applied to study differences in repair capacity in healthy exposed populations [44]. There are ethnic and geographical differences in the frequency distribution of genetic variants. Various technologies have been developed for low- and high-throughput genotyping. Additionally, markers of acquired susceptibility need to be considered, such as biomarkers of previous diseases or biomarkers of previous exposures such as epigenetic changes.

Biomarkers can also be used for the prediction of the clinical course and outcomes of disease under natural history or under treatment. Although these clinical uses are usually outside the scope of traditional aetiological research, this is a very rapidly expanding literature [45]–[47],[23] with major challenges. Although the current recommendations could apply to these uses, for tumor marker prognostic studies, the reader should refer to the REMARK guidelines [7].

Biomarker-based measurements are not, however, problem free. As in classical biomedical and epidemiological research, considering methodological issues concerning the design, conduct, analysis and interpretation of the results is essential to adequately address a research question [6]. In addition to the usual problems of bias and confounding that affect all clinical and epidemiological studies, particular issues when using biomarkers include (i) validity and reliability of biomarker measurements, (ii) special sources of bias, (iii) reverse causality and (iv) false positives as a result of multiple testing or selective reporting. To conceive relevant and valid studies, in biomarker-based research, we need an in-depth understanding and integration of methodological and substantive (i.e. biological, clinical and environmental) knowledge. Complete, accurate and transparent reporting of study design, methods, conduct and findings is required to allow the study to be fairly and adequately evaluated and summarized including avoidance of selective reporting of positive results [7]–[10]. Empirical evidence suggests that the results of the most highly cited biomarker studies across medicine almost consistently report larger effect estimates than those reported in subsequent meta-analyses [11]. Suboptimal reporting may also lead to inflated expectations on the translational potential and clinical utility of findings [12]. At the other end of the spectrum, false negatives are also a common problem [9], and they may result from limited sample size, poor study design or inappropriate laboratory assays [13].

The need for improved reporting of scientific research in general led to influential statements of recommendations such as CONSORT for randomized controlled trials [14],[15] and STrengthening Reporting of OBservational studies in Epidemiology (STROBE) statement [16]. The STROBE initiative was established in 2004 aiming at providing guidance on how to report observational research. The resultant STROBE statement was simultaneously published in several medical journals in 2007 [16],[17]. Its guidelines provide a user-friendly checklist of 22 items to be reported in epidemiological studies, with items specific to the three main study designs: cohort studies, case–control studies and cross-sectional studies. The STROBE statement has had an important impact. Its recommendations were adopted by several journals, and there is evidence that they have affected the style of result reporting [18]. However, there is also evidence of misuse of the STROBE statement [19].

Recent advances in molecular biology and the vast amount of data generated by high-throughput techniques (and consequent changes and improvement in terms of epidemiology, statistical analysis and study design) warrant implementing the STROBE recommendations specifically for molecular epidemiology studies. For a review of the state of the art of molecular epidemiology and the ensuing methodological problems, see [1]. Molecular tools (biomarkers) are also increasingly applied in epidemiology because of new and difficult issues that are addressed, such as the effects of chronic low-level exposures. While important discoveries of the past – such as the role of cholesterol or tobacco smoking – originated from studies with strong associations identified based on single measurements, there is now a challenge to identify weaker associations, and these require more accurate and sensitive tools. This increases the importance of a meticulous, comprehensive and transparent description of studies involving biomarkers.

Herein, we propose an extension of STROBE, i.e. STROBE for molecular epidemiology, STROBE-ME. The guidelines aim to provide an easy-to-use checklist of items that authors may use for reporting molecular epidemiology studies other than genetic association studies.

Recommendations already exist for genetic association studies, a field that has specific characteristics and requirements of reporting which have been included in a separate recent statement (STREGA, an extension of STROBE) [20]. There is some necessary overlap between the current guidelines and STREGA, insofar as ‘susceptibility biomarkers’ are included in the present recommendations. Communication of results of molecular epidemiology studies is a still underdeveloped field. This paper refers only to scientific communication of study results and does not address the ethical problem of communicating results to single individuals, see [21],[22].

## Aims and Use of the STROBE-ME Statement

The expected outcome of the present recommendations is an improvement in the reporting of results, such that the editors, reviewers of papers and the readers understand better what was actually done by the authors. STROBE-ME is expected to lead to more organized and transparent papers and to a better understanding of both the strengths and weaknesses of the studies in molecular epidemiology. Our recommendations do not dictate how studies should be performed nor do they serve as a basis to evaluate the quality of observational studies; they only try to help improve the reporting of research. The adoption of improved reporting standards may nevertheless have also an indirect benefit on the quality of study design.

The parent STROBE statement is a checklist of 22 items to be addressed when observational epidemiological studies are reported. The STROBE items cover different aspects of reporting a study: the title (one item), introduction (two items), methods (nine items), results (five items), discussion (four items) and funding of research (one item) [16]. The explanation and elaboration document of STROBE [17] explains these items in detail and provides good real-life examples in published works for their application.

The statement proposed here is intended to be an extension of the STROBE statement for molecular epidemiology studies. The present recommendations are intended only for those studies in which biomarkers are used as an explanatory variable; these include biomarkers of exposure/internal dose, biomarkers of early biological change and biomarkers of susceptibility (Box 1, and Figure 1). This set of biomarkers is used as measurable proxy for the process of the interaction between an external/endogenous agent and the body at different biological levels. Other study designs involving biomarkers are not covered by the present recommendations, including transitional studies of validation and reliability of measurement.

Some items belonging to the original STROBE checklist have been implemented for molecular epidemiology studies; other items have been added de novo to the original checklist. The 10 implemente items include issues on study design specificities in molecular epidemiology studies; description of relevant participant conditions at the time of sample collection; and particular statistical aspects if the biomarker measurements are introduced into statistical models. The seven new specific items added to the original STROBE checklist include biological sample collection, storage and processing; and the laboratory methods used for the analyses. The present extended checklist was developed as an extension of the STROBE checklist (Table 1). The recommendations are intended to complement the existing STROBE guidelines, not to replace them; therefore, all previously described items concerning observational studies such as cohort, case–control and cross-sectional studies apply to molecular epidemiological studies (when appropriate).

**Table 1:** The Strengthening the Reporting Observational studies in Epidemiology – Molecular Epidemiology (STROBE-ME) Reporting Recommendations: Extended from STROBE statement.

Item	Item number	STROBE Guidelines	Extension for Molecular Epidemiology Studies (STROBE-ME)
**Title and abstract**	1	(a) Indicate the study's design with a commonly used term in the title or the abstract	**ME-1** State the use of specific biomarker(s) in the title and/or in the abstract if they contribute substantially to the findings
		(b) Provide in the abstract an informative and balanced summary of what was done and what was found	
**Introduction**			
Background rationale	2	Explain the scientific background and rationale for the investigation being reported	**ME-2** Explain in the scientific background of the study how/why the specific biomarker(s) have been chosen, potentially among many others (e.g., others are studied but reported elsewhere, or not studied at all)
Objectives	3	State specific objectives, including any pre-specified hypotheses	**ME-3** *A priori* hypothesis: if one or more biomarkers are used as proxy measures, state the *a priori* hypothesis on the expected values of the biomarker(s)
**Methods**			
Study design	4	Present key elements of study design early in the paper	**ME-4** Describe the special study designs for molecular epidemiology (in particular nested case/control and case/cohort) and how they were implemented
*Biological sample collection*			**ME-4.1** Report on the setting of the biological sample collection; amount of sample; nature of collecting procedures; participant conditions; time between sample collection and relevant clinical or physiological endpoints.
*Biological sample storage*			**ME-4.2** Describe sample processing (centrifugation, timing, additives, etc).
*Biological sample processing*			**ME-4.3** Describe sample storage until biomarker analysis (storage, thawing, manipulation, etc).
*Biomarker biochemical characteristics*			**ME-4.4** Report the half-life of the biomarker, and chemical and physical characteristics (e.g., solubility).
Setting	5	Describe the setting, locations, and relevant dates, including periods of recruitment, exposure, follow-up, and data collection	
Participants	6	(a) Cohort study—Give the eligibility criteria, and the sources and methods of selection of participants. Describe methods of follow-upCase-control study—Give the eligibility criteria, and the sources and methods of case ascertainment and control selection. Give the rationale for the choice of cases and controlsCross-sectional study—Give the eligibility criteria, and the sources and methods of selection of participants	**ME-6** Report any habit, clinical conditions, physiological factor, or working or living condition that might affect the characteristics or concentrations of the biomarker
		(b) Cohort study—For matched studies, give matching criteria and number of exposed and unexposedCase-control study—For matched studies, give matching criteria and the number of controls per case	
Variables	7	Clearly define all outcomes, exposures, predictors, potential confounders, and effect modifiers. Give diagnostic criteria, if applicable	
Data source/measurement	8	For each variable of interest, give sources of data and details of methods of assessment (measurement).Describe comparability of assessment methods if there is more than one group	**ME-8** Laboratory methods: report type of assay used, detection limit, quantity of biological sample used, outliers, timing in the assay procedures (when applicable) and calibration procedures or any standard used
Bias	9	Describe any efforts to address potential sources of bias	
Study size	10	Explain how the study size was arrived at	
Quantitative variables	11	Explain how quantitative variables were handled in the analyses. If applicable, describe which groupings were chosen, and why	
Statistical methods	12	(a) Describe all statistical methods, including those used to control for confounding	**ME-12** Describe how biomarkers were introduced into statistical models
		(b) Describe any methods used to examine subgroups and interactions	
		(c) Explain how missing data were addressed	
		(d) Cohort study—If applicable, explain how loss to follow-up was addressedCase-control study—If applicable, explain how matching of cases and controls was addressedCross-sectional study—If applicable, describe analytical methods taking account of sampling strategy	
		(e) Describe any sensitivity analyses	
*Validity/reliability of measurement and internal/external validation*			**ME-12.1** Report on the validity and reliability of measurement of the biomarker(s) coming from the literature and any internal or external validation used in the study.
**Results**			
Participants	13	(a) Report the numbers of individuals at each stage of the study—e.g., numbers potentially eligible, examined for eligibility, confirmed eligible, included in the study, completing follow-up, and analysed	**ME-13** Give reason for loss of biological samples at each stage
		(b) Give reasons for non-participation at each stage	
		(c) Consider use of a flow diagram	
Descriptive data	14	(a) Give characteristics of study participants (e.g., demographic, clinical, social) and information on exposures and potential confounders	
		(b) Indicate the number of participants with missing data for each variable of interest	
		(c) Cohort study—Summarise follow-up time (e.g., average and total amount)	
*Distribution of biomarker measurement*			**ME-14.1** Give the distribution of the biomarker measurement (including mean, median, range, and variance)
Outcome data	15	Cohort study—Report numbers of outcome events or summary measures over timeCase-control study—Report numbers in each exposure category, or summary measures of exposureCross-sectional study—Report numbers of outcome events or summary measures	
Main results	16	(a) Give unadjusted estimates and, if applicable, confounder-adjusted estimates and their precision (e.g., 95% confidence interval).Make clear which confounders were adjusted for and why they were included	
		(b) Report category boundaries when continuous variables were categorized	
		(c) If relevant, consider translating estimates of relative risk into absolute risk for a meaningful time period	
Other analyses	17	Report other analyses done—e.g., analyses of subgroups and interactions, and sensitivity analyses	
**Discussion**			
Key results	18	Summarise key results with reference to study objectives	
Limitations	19	Discuss limitations of the study, taking into account sources of potential bias or imprecision. Discuss both direction and magnitude of any potential bias	**ME-19** Describe main limitations in laboratory procedures
Interpretation	20	Give a cautious overall interpretation of results considering objectives, limitations, multiplicity of analyses, results from similar studies, and other relevant evidence	**ME-20** Give an interpretation of results in terms of *a-priori* biological plausibility
Generalisability	21	Discuss the generalisability (external validity) of the study results	
**Other information**			
Funding	22	Give the source of funding and the role of the funders for the present study and, if applicable, for the original study on which the present article is based	
Ethics			**ME-22.1** Describe informed consent and approval from ethical committee(s). Specify whether samples were anonymous, anonymised or identifiable

The present statement contains a checklist of items for reporting molecular epidemiology studies (Table 1); some explanatory text referring to single item description; and some Boxes in which specific aspects of molecular epidemiology are briefly addressed for readers' reference. Although the current recommendations could apply also to biomarkers used for the prediction of clinical course and outcomes of disease, for tumour marker prognostic studies the reader should refer to the REMARK guidelines [7].

Concerning the uses of the present statement, additional details on how the parent STROBE statement was used can be found on the website (http://www.strobe-statement.org/). It is expected that the statement will be adopted and referred to by journals that publish molecular epidemiology papers, as well as by journals that publish clinical research in which biomarkers have an important role [23].

## Development of the STROBE-ME Statement

A multidisciplinary group of epidemiologists, biostatisticians and laboratory scientists (overall approximately 15 scientists) developed the current recommendations. Also, editors of several specialist journals were involved from the outset. The group met twice in London (UK) in 2008 and 2009, once in Turin (Italy) in 2009 and once in Łódź (Poland) in 2010; it sought external opinions from partners of the Environmental Cancer Risk, Nutrition, and Individual Susceptibility (ECNIS) European Network of Excellence – which was the initiator of the STROBE-ME initiative. Overall, the process lasted 3 years. While no formal process such as a Delphi consultation was used for development, consensus was built by circulating several versions of the statement within the group of developers and an external circle of potential users. In all, over 30 scientists were involved in the process.

## Checklist of Items

The items that should be considered when reporting molecular epidemiology studies are shown in Table 1 and available as supporting information. These items are similar to those that were originally recommended in STROBE, however, with modifications that are specific to molecular epidemiology. Later, we give a detailed description of each item. The purpose is not to suggest how to set up a research project but how to improve reporting of the research to allow readers (and reviewers) to better understand what was actually done by the researchers.

### ME-1 – State the use of biomarker(s) in the title and/or in the abstract if they contribute substantially to the findings

When one or more biomarkers are measured in an epidemiological study, it may be more informative reporting this in the title or at least in the abstract of the article. This helps the reader to identify immediately molecular epidemiology studies and ensures a correct indexing in electronic databases.

### ME-2 – Explain in the scientific background of the paper how/why the specific biomarker(s) have been chosen, potentially among many others

The process leading to the choice of one or more specific biomarkers for inclusion in a paper should be made clear in the Introduction. Background information and rationale for the choice of the specific biomarker(s) should be explicitly stated; also, how the biomarker is introduced in the study design should be made explicit (biomarker of exposure, internal dose, early biological change and susceptibility). It should also be clarified whether the biomarker is used as a proxy, and if so, what it is intended to be a proxy for.

### ME-3 – *A priori* hypothesis: if one or more biomarkers are used as proxy measures, state the a priori hypothesis on the expected values of the biomarker(s)

When stating the objective(s) of a study according to the STROBE guidelines [16], it might be helpful to state explicitly the a priori hypothesis on the expected values of the biomarker(s).

### ME-4 – Describe the special study designs for molecular epidemiology (in particular nested case–control and case–cohort) and how they were implemented

Study design details should be reported in the Methods section. For traditional designs such as case–control, cohort and cross-sectional studies, the STROBE recommendations can be followed, with extra care in reporting the biological sample collection integration within study design; for nested case–control and case–cohort studies, selection criteria for cases and controls, sampling frame and matching criteria should be reported with extra care, as they represent a main potential source of bias in these study designs (see Box 2). In addition to matching criteria for individuals, all methods used for selecting or matching biological samples (i.e. by storage time and by batch) should be reported. Also, it is recommended to describe briefly the cohort in which nested studies were implemented, in terms of description of the population, sampling, outcome ascertainment, follow-up period, number of subjects lost to follow-up and primary objective for which the cohort was established.

**Box 2. Specificities of Study Design for Molecular Epidemiology: Nested Case–Control Studies and Case–Cohort Studies**

Molecular epidemiology uses the same study designs as the general epidemiology, but some variants are more common. In particular, case–control studies nested in cohorts and case–cohort studies are frequently used to avoid extensive and costly measurements in large cohorts. In nested case–control studies derived from established cohorts, controls are usually matched for age and sex, and also for time variables related to sample collection and disease onset. The method of control selection in these studies is ‘incidence density’ sampling, and an incidence risk ratio is estimated. Controls may develop the disease of interest subsequently to the diagnosis of the case, but they represent the cohort set at risk of developing the disease when each case occurs [24]. The criteria for case inclusion and control matching and selection and their rationale should be reported [1].

In case–cohort studies, unmatched controls come from a sample of the cohort at inception without being matched to cases on time to outcome. The method for control selection in these studies is based only on the population at baseline, without regard to failure times, and a risk ratio is estimated [24],[1].

Both study designs share the important feature that cases and controls come from the same cohort study: recall bias is not of concern if exposure assessment was carried out before disease onset; nonparticipation bias is avoided because rapidly fatal cases have the same probability of inclusion as others; and reverse causation becomes less likely as biological samples were collected before the onset of the clinically documented disease. The nested case–control study tends to be more efficient than the case– cohort study in selecting controls to address confounding. In case–cohort studies, however, the same sample of controls can be compared to different samples of cases (thus different outcomes can be studied). Also, as the sub-cohort is a random sample of the whole cohort, prevalence of exposure can be estimated and external comparisons can be made.

The main concerns regarding nested case–control studies are that controls are not representative of the cohort population and they have few other uses, so the investment in biomarker analyses cannot be leveraged for other research. On the other hand, case–cohort studies rely on the assumption that exposure can be equally well measured in the sub-cohort as in the cases. However, three issues regarding biomarker validity make this assumption questionable: batch effects, the storage effect and freeze–thaw cycles. There are technological and staffing limits to how many samples can be analysed in one go so samples are run in batches or groups. Conditions of the analyses should not vary by batch, but it is clear that for many biomarker measurements this is not true, i.e. there are substantial batch effects (laboratory variation). Also, not all biomarker targets are stable at the usual storage temperature (−80°C), and when samples freeze and thaw, the pH and ionic balance of the liquid phase of the sample can be very different from the natural condition of the sample. Changes in pH and ionic balance can degrade biomarker targets. For these reasons, it may be necessary to include matching by length of storage, batch and freeze–thaw cycles [1].

### ME-4•1 – Report on the setting of the biological sample collection; amount of sample; nature of collecting procedures; participant conditions; time between sample collection and relevant clinical or physiological endpoints

An accurate description of the sample collection and shipment is necessary to enable the reader to evaluate potential sources of bias or errors in the biomarker measurement and for ensuring an appropriate reproducibility of the scientific experiment (see Box 3). The following items should be reported: (i) the setting of the biological sample collection (place, time of the day, time of the year, laboratories involved, personnel involved, etc.); (ii) amount/volume/size of sample(s); (iii) nature of the collecting procedure (anticoagulant involved, e.g. heparin, EDTA) (iv) if the participant is healthy, participant condition at the sample collection (fasting status, position, etc.) when appropriate; (v) if participants are not healthy individuals in stable physiological conditions, then report the relevant aspects of the health status and clinical conditions of the participants [24],[25]; (vi) in all instances, consider reporting the time between sample collection and relevant clinical or physiological endpoints that might have affected the characteristics or concentrations of the biomarker [26]. In particular, report any relevant characteristic of the participants, which might influence the biomarker levels in any known or unknown way. For example, position of the study subjects, such as orthostatism decreases plasma volume, so that proteins and cholesterol levels can be lowered by 5–15% relative to the supine position.

**Box 3. Collection, Handling and Storage of Biological Samples**

Several types of human biospecimens can be collected for carrying out molecular epidemiology studies. Blood samples may be stored as a whole or separated into sub-fractions and blood components (red blood cells, serum, plasma, buffy coat and white blood cell sub-fractions). White blood cells contained in the buffy coat are the most widely used source of DNA. Urine can be used as a solution of excreted parent compounds and metabolites to be measured, or as a source of exfoliated cells of the urinary tract. Collection and primary processing are performed accordingly. Other human tissue specimens used in molecular epidemiology studies include body fluids (i.e. cerebrospinal fluids), cell washes (i.e. buccal wash or swabs), epithelial smears, surgical material, nails and hair. Each step in collection, storage, thawing, manipulation and laboratory analysis can introduce errors that may lead to bias and variability. Random error, if evenly distributed in study subgroups, is likely to attenuate or eliminate differences. Systematic errors (e.g. differential clinical conditions, handling or storage of biological samples from cases and non-cases) may generate spurious associations.

Timing of collection often influences the true biomarker level. For example, hormones have hourly, daily or monthly cycles. Prolonged venipuncture can induce release of prolactin or increase white blood cell counts. A very narrow needle causes haemolysis. Several additives can be added to blood, e.g. metaphosphoric acid for vitamin C; anticoagulants such as heparin, EDTA or citrate are needed for plasma collection (i.e. not needed if only serum is collected). There may be disadvantages: heparin binds to many proteins and influences T-cell proliferation; EDTA interferes with cytogenetic analyses. Citrate-stabilized blood affords better quality of RNA and DNA than other anticoagulants. Other additives include protease inhibitors and RNAse inhibitors to avoid degradation of proteins and RNA, respectively.

The goals of proper sample storage are to ensure (i) standardized procedures for all phases; (ii) minimal loss or degrading of material (e.g. because of malfunctioning of freezers); (iii) optimal preservation of material; (iv) blinding, whenever appropriate; (v) easy access to the material when needed; (vi) easy matching of biological material with individual identity; (vii) respect of confidentiality; and (viii) anticipation of emergencies. Stability of the compounds to be measured depends on the type of measurement and temperature of storage: for example, fatty acids should be measured within 2 weeks when samples are stored at 4°C, within a few months when stored at −20°C, up to one year when stored at −80°C. A few studies have been conducted on the stability of different analytes, but the literature is far from being exhaustive.

Detailed information on all critical steps that might have altered the biological samples or influenced the final biomarker measurement should be identified and reported accordingly in the Methods section.

### ME-4•2 – Describe sample processing (centrifugation, timing, additives, etc.)

A comprehensive description of all steps of sample processing is needed in the Methods section to assess experimental reproducibility. This description ranges from manual handling of samples to specific machinery used for laboratory processing (see Box 3). When a well-established technique is used, the main process can be referred to by quoting the article where the technique is described and any variation from the initially described laboratory technique should be explicitly stated.

### ME-4•3 – Describe sample storage until biomarker analysis (storage, thawing, manipulation, etc.)

Particularly in nested case–control and case–cohort studies, biomarkers can be measured in biological samples stored for extended durations; sometimes, samples may have already undergone freeze–thaw cycles. As these processes can partially alter the biomarker values under examination, it is important to report in the Methods section any manipulation that the biological samples may have undergone, together with a detailed description of how the samples were stored.

### ME-4•4 – Report the half-life of the biomarker and chemical and physical characteristics (e.g. solubility)

For new biomarker(s) only, some basic biochemical information relevant to the interpretation of the measured values should be reported in the Methods section. This includes biochemical and biophysical characteristics that might be relevant when interpreting the results, such as half-life, solubility or lipophilicity.

### ME-6 – Report any habit, clinical condition, physiological factor, or working or living condition that might affect the characteristics or concentrations of the biomarker

Report any relevant characteristic of the participants, which might influence the biomarker levels in any known or unknown way [24]. For example, exposure to air pollution [27] or seasonality [28] might influence DNA adduct levels in healthy subjects; similarly, type of diet [29],[30] or amount of sunlight exposure [28],[31] might influence DNA damage biomarkers in healthy subjects.

### ME-8 – Laboratory methods: report type of assay used, detection limit, quantity of biological sample used, outliers, timing in the assay procedures (when applicable) and calibration procedures or any standard used

The methods used in the laboratory for biomarker analyses should be described in detail in a dedicated section of the Methods. Particular care should be taken to describe new or modified techniques, while for a well-established technique, the main process can be referred to by quoting the article where the technique is described, and any variation from the initially described laboratory technique should be explicitly stated. Any calibration procedures or external standards used in the laboratory (or for comparing data coming from different laboratories) should also be described. The definition of ‘outlier’ should be clearly given (for example, whether it is based on pathophysiological, technical or statistical grounds).

### ME-12 – Describe how biomarkers were introduced into statistical models

Usually, statistical methods that apply to biomarkers do not differ from those used in other branches of epidemiology and clinical research. Here, we mainly refer to specificities of biomarker research. When continuous variables are used (a very common occurrence for biomarkers), testing for linearity may be useful when the marker is used as a covariate, in addition to checking other statistical model assumptions when it is used as an outcome. Statistical manipulation of a variable derived from biomarker measurement values should be described in detail as for other variables included in the statistical models. Whether the variable is introduced as a continuous or categorical variable (and if categorical what criterion has been used for identifying cut-off points); whether extreme values have been excluded, and with which criteria; whether the original variable has been log transformed or manipulated in any other way; whether crude measurements or corrected/adjusted values (e.g. ratios to binding hormones and creatinine-adjusted values) were analysed; and how samples with nondetectable biomarker levels were dealt with (e.g. considered as zero, as the detection limit, as half of that level or imputed) should be clearly stated.

### ME-12•1 – Report on the validity and reliability of measurement of the biomarker(s) coming from the literature and any internal or external validation used in the study

Validity and reliability of biomarker(s) measurement should be reported when every specific biomarker is introduced (see Box 4). Measurement error has several components, and there is ambiguity on the use of the term, because ‘error’ encompasses both true ‘variations’ and ‘mistakes’. ‘Analytical’ measurement errors originate from the laboratory technique(s), including between-batch variation, while other sources of ‘pre-analytical error’ include variations in the individuals or the samples that are investigated [1]. Ideally, the inter-individual, intra-individual and inter-laboratory variations should be reported for each biomarker to enable the reader to understand the potential source of error for each specific biomarker. Literature-based reliability estimates should be properly referenced. When these figures are not available from the literature, this should also be stated. If aspects of the validity and reliability have been determined as part of the current study, the methods and process should be briefly stated. When a specific laboratory procedure or method for biomarker measurement has been standardized across laboratories for facilitating the comparability, this should be clearly stated [32],[33].

**Box 4. Biomarker Validity and Reliability**

To achieve an accurate estimate of the association between a biomarker and a disease, reliable and valid measurements of exposure, covariates (potential confounders and effect modifiers) and outcomes are needed [48]. Validity is defined as the (relative) lack of systematic measurement error when comparing the actual observation with a standard, which is a reference method representing the ‘truth’. While validity entails a ‘standard’, reliability (reproducibility and repeatability) concerns the extent to which any measuring procedure yields the same results in repeated experiments [49].

Validity and reliability are separate entities: a measurement may be perfectly reliable (reproducible in different laboratories and repeatable at different times), but consistently wrong, i.e. far away from the true value; conversely, another one can be unbiased on average, but unreliable if the measurements scatter widely around a true value. Both validity and reliability are important; however, as validity is often not measurable, reliability is sometimes used (incorrectly) as a surrogate.

Timing is also a relevant aspect: inferences about the meaning of biomarker measures are often strictly time specific, as time influences the results in several different ways [49]. For example, while DNA genetic variants are the same for each individual through one's life time, their epigenetic profile may change markedly over time.

Biomarker variability influences associations with the endpoint, thus needs to be assessed and reported upon. A single measure of a biomarker for one individual will be affected by (i) variability within subject (intra-subject); (ii) biological sample variation (i.e. variation depending on the frame of biological sample collection); and (iii) laboratory variation.

Intra-individual variation is sometimes so large that between-individual variation (usually the unit of interest) is hard to detect. A single biological measurement (assume that this is in the absence of laboratory variation) represents the biomarker level/status at a particular time. The biomarker may undergo diurnal, monthly, seasonal or longer variations, e.g. prolactin has a circadian rhythm, oestrogens vary through the menstrual cycle, biomarkers related to recent fruit and vegetable intakes may have seasonal variations. Other biomarkers are more stable, i.e. have less intra-individual variation, and thus, a single measure/sample is usually sufficient (such as mercury in hair, SNPs – single nucleotide polymorphisms). Variation in exposure to other compounds may have influence on the marker level. Intra-individual variability can be measured only if repeated samples from the same individual are collected [50]. Depending on the research question, a measure of a recent, short-term or instantaneous level may be desired (e.g. current CD4 count in a HIV patient), or an average level over a specified time interval (e.g. usual vitamin D level).

Biological sampling variation is related to the circumstances of biological sample collection. For example, hyperproliferation of colonic cells is extremely variable at different segments of the colon mucosa. Therefore, not only the intra-subject variation over time is important, because of the varying exposure to agents that induce cell proliferation, but also the measurements are strongly influenced by how and where the mucosa is sampled from.

Laboratory measurements can have many sources of error, in particular two general classes of laboratory errors: those that occur between analytical batches and those that occur within the batches. Handling, processing and storing of specimens may contribute to errors. Laboratory procedures need to be in place to minimize such variation and avoid biases. Quality control procedures such as the inclusion of laboratory quality control samples and blinded split samples are used to assess the extent of these errors. There should be no identifiers that relate the sample to any other characteristics of the individual from whom it came and in particular of their disease status or any other factor.

The errors of biomarker measurement may have different impact depending on their error distribution. If the epidemiological study has been conducted blindly, i.e. the laboratory analyses have been carried out with no knowledge of the exposed/unexposed or diseased/healthy status of the subjects, the measurement error is expected to be evenly distributed across strata of exposure or disease. However, this is true only if the error is equally distributed across the scale of the exposure. This kind of misclassification leads to underestimation of the risk ratio because of a ‘blurring’ of the relationship between exposure and disease. Both underestimation and overestimation of the association of interest may occur when misclassification is not evenly distributed across the study variables [51]. Individuals with extreme biomarker levels may be excluded, or sensitivity analyses are carried out with and without them to check whether they overly influence the general findings.

The most important single measure of biomarker reliability is the intra-class correlation coefficient (ICC). This is a quantitative measurement of the between-person variance divided by the total (between plus within-subject) variance [52]. It describes how strongly measurements taken in the same subject resemble each other in comparison with the inter-individual variance.

Biomarker measurement validation is particularly important when a new biomarker is described. Without information on measurement error, intra-individual variation and inter-individual variation biomarker studies are uninterpretable. Also, variation by batch is usually very relevant and may create artifactual relationships [34]. For more detailed presentation of validity and reliability issues, see Box 4.

Besides validity and reliability of biomarker measurements, it is increasingly recognized that the study results are likely to be more credible when they have been reproduced by some additional validation process, either internally (e.g. by cross-validation) or preferably with external independent validation in samples that are totally different from those where the biomarker was first tested [35]. All attempts at internal and external validation carried out by the authors should be reported in detail in the Methods section, and the respective results should be shown in the Results section.

### ME-13 – Give reasons for the loss of biological samples at each stage

Loss of specimens, non-evaluable samples (because of poor quality) or assay failures are common occurrences. When some samples are not included in the final analysis because of problems in sample quality, quantity, availability, timing of sample collection or technical failure give detailed reasons. This will help in tracking the final sample size and the reasons for sample exclusions.

### ME-14•1 – Give the distribution of the biomarker measurement (including mean, median, range and variance)

An appropriate description of the biomarker measurement distribution is of help for interpreting results and for comparing similar biomarker measurements by other scientists. It also often facilitates the biological interpretation of the results. A graph of the full distribution may be useful (when relevant, also by exposure status or case/control status).

### ME-19 – Describe main limitations in laboratory procedures

Potential and actual limitations met in laboratory procedures should be described in detail in the Discussion. It may be helpful also to report whether the limitation would likely have introduced a random or systematic error and, if systematic, to suggest in which direction this might have biased the results. Validation of results of biomarker studies is of major importance, and the discussion should address whether any validation procedure was used in the study [36].

### ME-20 – Give an interpretation of results in terms of a priori biological plausibility. Results should be interpreted in the light of the mechanism(s)

Of action of the biomarker(s) and of the a priori hypothesis, thus offering a biologically plausible interpretation. It may be useful to stress the added value of the biomarker(s) in explicating the biological mechanism underlying the association reported.

### ME-22•1 – Describe informed consent and approval from ethical committee(s). Specify whether samples were anonymous, anonymized or identifiable

Molecular epidemiology poses special ethical issues that are summarized in Box 5.

**Box 5. Ethical Considerations**

Legal issues related to the use of stored human biological material are contained in a European guideline issued by the Council of Europe (http://www.coe.int). In the United States, a useful website is http://nih.gov/sigs/bioethics. When incorporating biospecimen-derived measurements, the following requirements should be met: follow respectful protocols in eliciting information; avoid harm to participants; secure proper informed consent, manage anonymization of interlinking databases; establish confidentiality and security safeguards; develop proper responses to requests for personal data by various parties; devise sound data access, ownership and intellectual property policies; be clear about whether and how individuals will be informed of findings that might be medically helpful for them; and arrange supervision by research ethics and privacy protection bodies [53].

Clearly, each of these requirements would need extensive comments. In particular, how ‘broad’ should the consent be? On the one hand, a broad consent (e.g. ‘the biological samples will be used for the identification of gene variants that may predispose to chronic diseases’) implies a greater freedom of the researcher, who is not obliged to collect further consent forms each time a new gene is investigated. On the other hand, such a generic informed consent form explains very little to the recruitees.

The concept of informed consent was initially formulated in the Declaration of Helsinki in 1964, with the latest revision in 2000 (http://www.wma.net). Recent developments in molecular epidemiology tend to overcome the conflict between ‘broad’ and ‘narrow’ consent forms, introducing the idea of a ‘two-level consent’, i.e. a relatively broad procedure at first, followed by a more specific and detailed approach when studies on single genes/biomarkers are conducted.

For example, there is a broad agreement that low-penetrant variants that are common in the general population and are associated with a slight increase in the risk (interacting with environmental exposures) should not be subject to strict rules as far as ethical implications are concerned. In fact, knowledge of presence or absence of a single allele involved in metabolic pathways neither allows the carrier to modify her/his risk profile substantially nor allows the researcher to identify other members of the family, which would violate confidentiality. The case of highly penetrant gene variants is different: e.g., the identification of the carrier of a rare mutation allows the researchers to identify other family members possibly affected, with potential detrimental effects (e.g. on insurance policies).

The same reasoning applies to biomarkers. The majority of biomarkers used in observational epidemiological research are of little utility to the subjects participating in the research, when taken alone. This is particularly true for the biomarkers of exposure, but also some biomarkers of early biological change/effect may not be meaningful when extrapolated from the research context; for example, DNA adduct level is difficult to interpret at a personal level. Researchers should have a clear view of the practical implications of testing for the study subjects, and in particular what to do in each of these situations: when no effective treatment is possible; when treatment is available with close balance of favourable/unfavourable effects; and effective treatment is available with scarce unfavourable effects. Similar considerations apply to biomarkers, which can be weakly or strongly associated with diseases and less or more associated with family history.

Anonymization of information is another difficult issue. First, there is a problem of definitions: ‘identifiable’ is a sample with name or social security number on it; ‘coded’ is a sample with a code that allows relatively easy identification of the person; ‘encrypted’ is a sample with a code that does not allow easy identification of the person, but this is possible with extra effort; finally, ‘anonymous’ is a sample for which there is no possibility of linking to a person. Clearly, a really anonymous collection of samples is of very little use for epidemiological research, which is based on follow-up and linkage of laboratory data and health-related data.

## Discussion

Transparent reporting is essential in epidemiology as in science in general, and in molecular epidemiology in particular. Given that the use of biomarkers has raised great expectations in terms of potential elucidation of disease aetiology and pathogenesis, it is important to raise awareness on the intrinsic limitations of biomarker measurements. In particular, measurement error is a common problem and can cause both false-negative and false positive results [9]. Also, the lack of a formal study design may substantially impair the interpretation of the results, and selective reporting of results can be detrimental.

The present STROBE-ME checklist should strengthen primarily the reporting and interpretability of molecular epidemiology studies, if used widely and systematically. It has been developed based on two strong foundations: (i) the well-established STROBE collaboration and the related statement and (ii) an ECNIS working group formed by epidemiologists, biostatisticians and laboratory scientists with extensive experience in the field of molecular epidemiology and biomarker analyses.

We hope that these guidelines will improve the quality of reporting of molecular epidemiology and other biomarker based research, including studies conducted within the growing number of biobanks and of biomonitoring projects.

The ethical duty of researchers includes reporting findings with accuracy, completeness and transparency, and in sufficient detail to allow the scientific community to consider them adequately, assess their strengths and weaknesses and make fair comparisons. Well-reported published studies can contribute to and be summarized with an evidence-based approach in an appropriate manner (i.e. on sound scientific grounds) to arrive at unbiased conclusions that lead to better knowledge and the advancement of citizens' health [37],[38].

Finally, we would like to stress that these recommendations, as the original STROBE statement and other guidelines on reporting research [7],[14],[16],[20], are evolving documents requiring continuous feedback, reassessment and refinement. The STROBE-ME guidelines will be published on the STROBE website (http://www.strobe-statement.org) where a forum for discussion and improvement of the checklist and related material will be available.

Guidance documents should also be appraised for their eventual impact. The EQUATOR initiative [39]–[41] has found that only 17% of the surveyed guideline developers performed a formal evaluation of the impact. We will engage journal editors in attempts to evaluate the impact of the present statement in the long run.

## Supporting Information

Table S1 The Strengthening the Reporting of Observational studies in Epidemiology – Molecular Epidemiology (STROBE-ME) Reporting Recommendations: Extended from STROBE statement.
(DOC)
