# Supplementary material for: STrengthening the Reporting of OBservational studies in Epidemiology – Molecular Epidemiology (STROBE-ME): An Extension of the STROBE Statement
Source: PLoS Med. 2011 Oct 25;8(10):e1001117. doi: 10.1371/journal.pmed.1001117 (PMC3201942; doi:10.1371/journal.pmed.1001117)
Supplement: Table S1 — The Strengthening the Reporting of Observational studies in Epidemiology – Molecular Epidemiology (STROBE-ME) Reporting Recommendations: Extended from STROBE statement. (DOC) [file pmed.1001117.s001.doc]

**Table S1**

(Gallo et al. [2011] PLoS Med; doi:10.1371/journal.pmed.1001117)

| The Strengthening the Reporting Observational studies in Epidemiology – Molecular Epidemiology (STROBE-ME) Reporting Recommendations: Extended from STROBE statement | | | |
| --- | --- | --- | --- |
| **Item** | **Item number** | **STROBE Guidelines** | **Extension for Molecular Epidemiology Studies (STROBE-ME)** |
| **Title and abstract** | 1 | (a) Indicate the study’s design with a commonly used term in the title or the abstract | **ME-1** State the use of specific biomarker(s) in the title and/or in the abstract if they contribute substantially to the findings |
|  |  | (b) Provide in the abstract an informative and balanced summary of what was done and what was found |  |
| **Introduction** |  |  |  |
| Background rationale | 2 | Explain the scientific background and rationale for the investigation being reported | **ME-2** Explain in the scientific background of the study how/why the specific biomarker(s) have been chosen, potentially among many others (e.g., others are studied but reported elsewhere, or not studied at all) |
| Objectives | 3 | State specific objectives, including any pre-specified hypotheses | **ME-3** *A priori* hypothesis: if one or more biomarkers are used as proxy measures, state the *a priori* hypothesis on the expected values of the biomarker(s) |
| **Methods** |  |  |  |
| Study design | 4 | Present key elements of study design early in the paper | **ME-4** Describe the special study designs for molecular epidemiology (in particular nested case/control and case/cohort) and how they were implemented |
| *Biological sample collection* |  |  | **ME-4.1** Report on the setting of the biological sample collection; amount of sample; nature of collecting procedures; participant conditions; time between sample collection and relevant clinical or physiological endpoints. |
| *Biological sample storage* |  |  | **ME-4.2** Describe sample processing (centrifugation, timing, additives, etc). |
| *Biological sample processing* |  |  | **ME-4.3** Describe sample storage until biomarker analysis (storage, thawing, manipulation, etc). |
| *Biomarker biochemical characteristics* |  |  | **ME-4.4** Report the half-life of the biomarker, and chemical and physical characteristics (e.g., solubility). |
| Setting | 5 | Describe the setting, locations, and relevant dates, including periods of recruitment, exposure, follow-up, and data collection |  |
| Participants | 6 | (a) Cohort study—Give the eligibility criteria, and the sources and methods of selection of participants. Describe methods of  follow-up  Case-control study—Give the eligibility criteria, and the sources and methods of case ascertainment and control selection. Give  the rationale for the choice of cases and controls  Cross-sectional study—Give the eligibility criteria, and the sources and methods of selection of participants | **ME-6** Report any habit, clinical conditions, physiological factor, or working or living condition that might affect the characteristics or concentrations of the biomarker |
|  |  | (b) Cohort study—For matched studies, give matching criteria and number of exposed and unexposed  Case-control study—For matched studies, give matching criteria and the number of controls per case |  |
| Variables | 7 | Clearly define all outcomes, exposures, predictors, potential confounders, and effect modifiers. Give diagnostic criteria, if applicable |  |
| Data source/measurement | 8 | For each variable of interest, give sources of data and details of methods of assessment (measurement).  Describe comparability of assessment methods if there is more than one group | **ME-8** Laboratory methods: report type of assay used, detection limit, quantity of biological sample used, outliers, timing in the assay procedures (when applicable) and calibration procedures or any standard used |
| Bias | 9 | Describe any efforts to address potential sources of bias |  |
| Study size | 10 | Explain how the study size was arrived at |  |
| Quantitative variables | 11 | Explain how quantitative variables were handled in the analyses. If applicable, describe which groupings were chosen, and why |  |
| Statistical methods | 12 | (a) Describe all statistical methods, including those used to control for confounding | **ME-12** Describe how biomarkers were introduced into statistical models |
|  |  | (b) Describe any methods used to examine subgroups and interactions |  |
|  |  | (c) Explain how missing data were addressed |  |
|  |  | (d) Cohort study—If applicable, explain how loss to follow-up was addressed  Case-control study—If applicable, explain how matching of cases and controls was addressed  Cross-sectional study—If applicable, describe analytical methods taking account of sampling strategy |  |
|  |  | (e) Describe any sensitivity analyses |  |
| *Validity/reliability of measurement and internal/external validation* |  |  | **ME-12.1** Report on the validity and reliability of measurement of the biomarker(s) coming from the literature and any internal or external validation used in the study. |
| **Results** |  |  |  |
| Participants | 13 | (a) Report the numbers of individuals at each stage of the study—e.g., numbers potentially eligible, examined for eligibility, con-  firmed eligible, included in the study, completing follow-up, and analysed | **ME-13** Give reason for loss of biological samples at each stage |
|  |  | (b) Give reasons for non-participation at each stage |  |
|  |  | (c) Consider use of a flow diagram |  |
| Descriptive data | 14 | (a) Give characteristics of study participants (e.g., demographic, clinical, social) and information on exposures and potential con-  founders |  |
|  |  | (b) Indicate the number of participants with missing data for each variable of interest |  |
|  |  | (c) Cohort study—Summarise follow-up time (e.g., average and total amount) |  |
| *Distribution of biomarker measurement* |  |  | **ME-14.1** Give the distribution of the biomarker measurement (including mean, median, range, and variance) |
| Outcome data | 15 | Cohort study—Report numbers of outcome events or summary measures over time  Case-control study—Report numbers in each exposure category, or summary measures of exposure  Cross-sectional study—Report numbers of outcome events or summary measures |  |
| Main results | 16 | (a) Give unadjusted estimates and, if applicable, confounder-adjusted estimates and their precision (e.g., 95% confidence interval).  Make clear which confounders were adjusted for and why they were included |  |
|  |  | (b) Report category boundaries when continuous variables were categorized |  |
|  |  | (c) If relevant, consider translating estimates of relative risk into absolute risk for a meaningful time period |  |
| Other analyses | 17 | Report other analyses done—e.g., analyses of subgroups and interactions, and sensitivity analyses |  |
| **Discussion** |  |  |  |
| Key results | 18 | Summarise key results with reference to study objectives |  |
| Limitations | 19 | Discuss limitations of the study, taking into account sources of potential bias or imprecision. Discuss both direction and magnitude  of any potential bias | **ME-19** Describe main limitations in laboratory procedures |
| Interpretation | 20 | Give a cautious overall interpretation of results considering objectives, limitations, multiplicity of analyses, results from similar studies, and other relevant evidence | **ME-20** Give an interpretation of results in terms of *a-priori* biological plausibility |
| Generalisability | 21 | Discuss the generalisability (external validity) of the study results |  |
| **Other information** |  |  |  |
| Funding | 22 | Give the source of funding and the role of the funders for the present study and, if applicable, for the original study on which the  present article is based |  |
| Ethics |  |  | **ME-22.1** Describe informed consent and approval from ethical committee(s). Specify whether samples were anonymous, anonymised or identifiable |
